# ISMB 2022 proceedings

**DOI:** 10.1093/bioinformatics/btac305

**Published:** 2022-06-27

**Authors:** Christophe Dessimoz, Sushmita Roy

**Affiliations:** Department of Computational Biology, University of Lausanne, Lausanne 1015, Switzerland; SIB Swiss Institute of Bioinformatics, Lausanne 1015, Switzerland; Department of Genetics, Evolution and Environment, University College London, London WC1E 6BT, UK; Department of Computer Science, University College London, London WC1E 6BT, UK; Wisconsin Institute for Discovery, University of Wisconsin-Madison, Madison, WI 53715, USA; Department of Biostatistics and Medical Informatics, University of Wisconsin-Madison, Madison, WI 53792, USA

This special issue of Bioinformatics serves as the proceedings of the 30th annual conference on Intelligent Systems for Molecular Biology (ISMB), which took place on July 10–14, 2022, in Madison, WI, USA. ISMB is the leading international forum for presenting new research results, disseminating methods and techniques and facilitating discussions among leading researchers, practitioners and students in the field. In addition, ISMB is the flagship conference of the International Society for Computational Biology. Due to the worldwide COVID-19 pandemic, the ISMB 2022 meeting was run as a hybrid conference, with online participants from all around the world complementing the on-site participants.

The papers published in this volume were selected from 243 submitted full-length papers featuring original research. The submitted papers were thoroughly reviewed with each paper receiving 4.08 reviews on average. For the review purpose, the submitted manuscripts were assigned to one of the 11 scientific areas according to the authors’ preference and research topic, allowing for minor adjustments to avoid conflicts of interest. In addition to selecting one of the 11 areas, the authors could also designate a particular Community of Special Interest (COSI; Table 1) that would provide the best forum for the presentation of their paper. The 11 research areas covered a broad spectrum of topics (Table 2) and also included a special General Computational Biology area intended for submissions on emerging topics or for those manuscripts that did not fit well in other reviewing areas. This year we also sought papers in a new area, equity-focused research.

**Table 1.** COSI distribution of accepted ISMB 2022 proceedings papers

COSI	Number of papers
3D-SIG	2
Bio-ontologies	2
CAMDA	2
CompMS	1
Education	2
EvolCompGen	7
Function	1
HiTSeq	9
iRNA	3
Microbiome	2
MLCSB	7
NetBio	2
RegSys	2
TransMed	5
VarI	1

**Table 2.** Thematic areas of ISMB 2022

Area	Area Chairs	Submissions	Accepted papers	Acceptance rate (%)
Bioinformatics Education	Nicola Mulder	2	2	100
Bioinformatics of Microbes and Microbiomes	Robert Beiko and Hélène Touzet	17	3	17.6
Biomedical Informatics	Barbara Engelhardt and Maria Secrier	45	6	13.3
Evolutionary, Comparative and Population Genomics	Lars Arvestad and Céline Scornavacca	20	4	20.0
Genome Privacy and Security	Hyunghoon Cho	4	1	25
Genome Sequence Analysis	Carl Kingsford and Rob Patro	33	8	24.2
Macromolecular Sequence, Structure, and Function	Yann Ponty and Jérôme Waldispühl	32	7	21.9
Regulatory and Functional Genomics	Jian Ma and Saurabh Sinha	36	5	13.9
Systems Biology and Networks	Tijana Milenkovic and Marinka Zitnik	28	6	21.4
General Computational Biology	Mohammed El-Kebir and Su-In Lee	25	5	20
Equity-focused Research	Casey Greene	1	0	0

*Note*: The table lists the Area Chairs for each theme, the number of reviewed papers, the number of accepted papers and the acceptance rate for each area.

The reviewing of the submissions was overseen by the Senior Program Committee (SPC), which included the Proceedings Chairs (this editorial’s authors) and Area Chairs (AC, listed in Table 2). Several of the ACs were nominated by COSIs or by the ISMB Steering Committee. Members of the SPC were responsible for recruiting the Program Committee. Reviewers (Program Committee Members and sub-reviewers recruited by them) judged the papers based on the novelty of computational approaches, the relevance of biological questions, the importance of biological insights, clarity of presentation, correctness and completeness of the study and expected impact. After submitting their reviews, the reviewers had the opportunity to discuss the papers and refine the scores. The ACs facilitated these discussions, oversaw the review process and made sure reviews were detailed and consistent with the overall decision. Final acceptance decisions were made by the entire SPC.

Throughout the reviewing process, we followed a stringent policy of guarding conflicts of interest. Submissions that had any association with an Area Chair were reassigned to a different area. Care was taken not to assign papers to reviewers with a perceived conflict either (e.g. co-authorship or same institution). The definition of conflict was defined as broadly as possible—including collaborators (present and past few years), same institution (present, past few years and planned future moves), family relations, advisees/advisors, as well as any personal conflicts that could cause the appearance of a conflict of interest, or that could genuinely interfere with objective reviewing. Finally, as Proceedings Chairs, we refrained from submitting papers to the conference.

Among the 243 submissions, 48 were accepted for presentation at ISMB 2022 and publication in the proceedings, conditioned on revisions properly addressing the comments of the reviewers. In a few cases, the authors had to be reminded to release their source code alongside their manuscript. This year, all 48 conditionally accepted papers were revised and subsequently judged to have properly addressed the concerns of the reviewers and were accepted for the conference proceedings, resulting in a 19.8% acceptance rate overall. The acceptance rates for individual areas are shown in Table 2. Accepted papers were assigned to COSIs based on the preferences of both authors and COSI organizers (Table 1).

We are deeply grateful to the Area Chairs, the 304 members of the Program Committee and the 270 sub-reviewers for their outstanding efforts in conducting thorough and timely reviews. Their contribution is at the core of the scientific quality of the conference. We also thank Steven Leard, Diane Kovats and Seth Munholland for their support, guidance and handling logistical questions and all the other members of the ISMB Steering Committee for their expert advice and supervision. We also thank the team at Oxford University Press for producing this special proceedings volume.

We also thank all the authors for submitting their work. These proceedings would not be possible without the scientific ingenuity of the contributors of all the papers. We recognize that, despite our best efforts, the selection process is necessarily imperfect, and some outstanding work will have been missed. Nonetheless, we hope that all authors received helpful feedback on their work. Finally, we want to thank all the keynote speakers, presenters, and all conference participants.

Thank you all for making this meeting possible and the entire ISMB community to continue to thrive.

## Data Availability

No new data were generated or analysed in support of this research.

## Funding

This work was supported by the James McDonnell Foundation to S.R.; Swiss National Science Foundation grants (205085 and 186397 to C.D.).

*Conflict of Interest*: none declared.

